# The value of periportal hyperintensity sign from gadobenate dimeglumine-enhanced hepatobiliary phase MRI for predicting clinical outcomes in patients with decompensated cirrhosis

**DOI:** 10.1186/s13244-024-01629-4

**Published:** 2024-02-27

**Authors:** Lanqing Cong, Yan Deng, Shuo Cai, Gongzheng Wang, Xinya Zhao, Jingzhen He, Songbo Zhao, Li Wang

**Affiliations:** 1 Department of Radiology, Shandong Provincial Hospital Affiliated to Shandong First Medical University, Jinan, Shandong Province 250021 China; 2 Department of Radiology, Qilu Hospital of Shandong University, Jinan, Shandong Province, 250012 China; 3 MRI Department, Shandong Provincial Hospital Heze Hospital, Heze, Shandong Province, 274031 China; 4 Department of Radiology, Shandong Provincial Hospital, Shandong University, Jinan, Shandong Province, 250021 China; 5 Department of Central Laboratory, Shandong Provincial Hospital Affiliated to Shandong University, Jinan, Shandong Province, China; 6 Shandong Provincial Engineering and Technological Research Center for Liver Diseases Prevention and Control, Jinan, Shandong Province, China; 7 Department of Health Management Center, Shandong Provincial Hospital Affiliated to Shandong First Medical University, Jinan, Shandong Province 250021 China

**Keywords:** Gadobenate dimeglumine, Periportal hyperintensity sign, Liver cirrhosis, Magnetic resonance imaging, Clinical outcomes

## Abstract

**Objectives:**

To determine the value of periportal hyperintensity sign from gadobenate dimeglumine (Gd-BOPTA)-enhanced hepatobiliary phase (HBP) magnetic resonance imaging (MRI) for predicting clinical outcomes in patients with decompensated cirrhosis.

**Methods:**

A total of 199 cirrhotic patients who underwent Gd-BOPTA-enhanced MRI were divided into control group (*n*  =  56) and decompensated cirrhosis group (*n*  =  143). The presence of periportal hyperintensity sign on HBP MRI was recorded. The Cox regression model was used to investigate the association between periportal hyperintensity sign and clinical outcomes.

**Results:**

There was a significant difference in the frequency of periportal hyperintensity sign on HBP between compensated and decompensated cirrhotic patients (*p*  <  0.05). After a median follow-up of 29.0 months (range, 1.0–90.0 months), nine out of 143 patients (6.2%) with decompensated cirrhosis died. Periportal hyperintensity sign on HBP MRI was a significant risk factor for death (hazard ratio (HR)  =  23.677; 95% confidence interval (CI)  =  4.759–117.788; *p*  =  0.0001), with an area under the curve (AUC) of 0.844 (95% CI  =  0.774–0.899). Thirty patients (20.9%) developed further decompensation. Periportal hyperintensity sign on HBP MRI was also a significant risk factor for further decompensation (HR  =  2.594; 95% CI  =  1.140–5.903; *p*  =  0.023).

**Conclusions:**

Periportal hyperintensity sign from Gd-BOPTA-enhanced HBP MRI is valuable for predicting clinical outcomes in patients with decompensated cirrhosis.

**Critical relevance statement:**

Periportal hyperintensity sign from gadobenate dimeglumine-enhanced hepatobiliary phase magnetic resonance imaging is a new noninvasive method to predict clinical outcomes in patients with decompensated cirrhosis.

**Key points:**

• There was a significant difference in the frequency of periportal hyperintensity sign on HBP between compensated and decompensated cirrhotic patients.

• Periportal hyperintensity sign on the hepatobiliary phase was a significant risk factor for death in patients with decompensated cirrhosis.

• Periportal hyperintensity sign on the hepatobiliary phase was a significant risk factor for further decompensation in patients with decompensated cirrhosis.

**Graphical Abstract:**

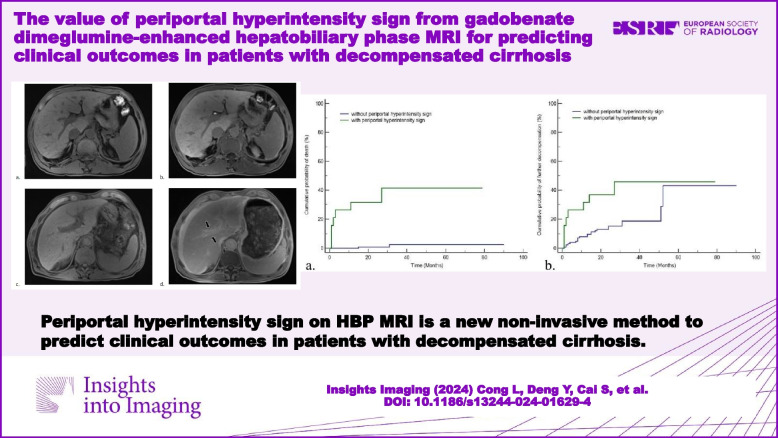

## Introduction

Decompensated cirrhosis is determined by the presence of ascites, hepatic encephalopathy, variceal bleeding, or other events [1, 2]. Following the first appearance of any of these symptoms, the disease usually progresses rapidly, and the patients are at a high risk of mortality [3]. The 5-year survival rate in decompensated cirrhotic patients is about 14 to 35%, which is shorter than that in compensated cirrhotic patients [2, 4]. Besides a significant mortality rate, decompensated cirrhosis is associated with considerable cost of treatment and patient suffering [5].

The Child–Pugh and model for end-stage liver disease (MELD) scores are well-recognized prognostic models of decompensated cirrhosis [6–8]. The advantage of these two scores is that they can be readily determined based on clinical and laboratory information [9]. Despite advances in the clinical outcomes of these methods for non-invasive liver assessment, their drawbacks have limited their extensive use. For example, there is no evidence that the cut-off levels for the Child–Pugh score are optimal [10], and the levels of creatinine and bilirubin included in the MELD score are easily altered by therapeutic interventions, hemolysis, or sepsis [11]. Therefore, new tools are needed to evaluate the prognosis in patients with decompensated cirrhosis.

Gadobenate dimeglumine (Gd-BOPTA) is a commonly used hepatocyte-specific contrast agent, which is utilized to characterize focal liver lesions [12]. Our previous studies have determined that Gd-BOPTA-enhanced hepatobiliary phase (HBP) magnetic resonance imaging (MRI) is a good approach for assessing liver function and clinical outcomes in cirrhotic patients [13, 14]. Gd-BOPTA-enhanced portal vein imaging on HBP can predict the prognosis in chronic liver disease patients [15]. Periportal hyperintensity sign on HBP is defined as enhancement manifested as a periportal ring that surrounds the portal veins and has been confirmed to be a useful indicator for predicting advanced liver fibrosis [16, 17]. However, no study has reported on the value of periportal hyperintensity sign on HBP for predicting clinical outcomes in patients with decompensated cirrhosis.

Therefore, the present study estimated the value of periportal hyperintensity sign from Gd-BOPTA-enhanced HBP MRI for predicting clinical outcomes in patients with decompensated cirrhosis.

## Materials and methods

### Patients

Patients with decompensated cirrhosis who underwent a Gd-BOPTA-enhanced liver MRI examination between November 2012 and December 2021 were retrospectively enrolled in this study. Cirrhosis was defined based on pathologic or clinical evidence, including nodularity/splenomegaly on liver imaging and/or thrombocytopenia [1]. Hepatic decompensation was identified by the presence of ascites, variceal bleeding, or hepatic encephalopathy (HE) [18, 19]. Exclusion criteria were as follows: the presence of malignancy, acute hepatitis, surgery involving the biliary tract, insufficient imaging quality, missing biochemical parameters, loss to follow-up, or renal impairment. Patients who underwent liver transplant surgery were withdrawn from the study. Overall, 56 compensated cirrhotic patients were included in the control group and 143 patients (91 men and 52 women) were included in the decompensated cirrhosis group. Decompensated cirrhotic patients were categorized as Child–Pugh A (*n*  =  23), Child–Pugh B (*n*  =  67), and Child–Pugh C (*n*  =  53) in accordance with their clinical manifestations.

Serum markers tested within 2 weeks of MRI were obtained using electronic medical records [13]. Outcomes, including death and further decompensation, were followed up in all patients. Follow-up time was the period from the first MRI to the time of the event or the end of the follow-up. Further decompensation was defined as recurrent acute variceal bleeding, refractory ascites, recurrent HE, spontaneous bacterial peritonitis, hepatorenal syndrome, acute-on-chronic liver failure (ACLF), and liver-related death [20, 21].

### MRI

All patients were examined on a 3-T MR scanner (MAGNETOM Verio or Prisma, Siemens). The liver protocols included pre- and post-contrast T1-weighted sequences. Dynamic sequences were obtained in 20 s (arterial phase), 50 s (late arterial phase), 80 s (portal venous phase), and 90 min (HBP) after contrast media injection at a concentration of 0.05 mmol/kg (0.1 mL/kg) of body weight followed by a 20-mL saline flush. Image parameters were as follows: repetition time, 3.31 ms; echo time, 1.3 ms; slice thickness, 3 mm; number of partitions, 72; matrix, 182 × 320; flip angle, 9°; and acquisition time, 17 s.

### Imaging analysis

Two radiologists (observers 1 and 2, with 10 and 13 years of experience, respectively), blinded to patients’ clinical history, independently reviewed all images [13]. Disagreements were resolved by discussion and achieving consensus. The presence of periportal hyperintensity sign on HBP was recorded. Periportal hyperintensity sign was defined as a periportal ring or tramline enhancement around the intrahepatic portal veins present within several (more than one) hepatic segments [16, 22].

### Statistical analysis

Student’s *t* test or Mann–Whitney *U* test was used for the comparison of the two groups. Interobserver agreement of categorical variables was estimated using Cohen’s kappa (*κ*) statistics. The Cox regression model was generated to identify factors related to death and further decompensation in patients with decompensated cirrhosis. Performance was assessed using the area under the curve (AUC). Then, the cumulative incidences of death and further decompensation were calculated using the Kaplan–Meier method. *p*  <  0.05 was considered significant. SPSS Statistics (version 25.0, IBM) and MedCalc (version 15.6.1, MedCalc Software) were used for all statistical analysis.

## Results

### Study sample characteristics

The median age of the recruited patients was 51 years. Hepatitis B virus (*n*  =  137, 68.8%) was the most common cause of cirrhosis, followed by alcohol use (*n*  =  21, 10.6%), cryptogenic disease (*n*  =  14, 7.0%), autoimmune liver disease (*n*  =  11, 5.5%), cholestasis (*n*  =  7, 3.5%), hepatitis C virus (*n*  =  5, 2.5%), and drug use (*n*  =  4, 2.0%). These patients were categorized as Child–Pugh A (58 of 199; 29.1%), Child–Pugh B (87 of 199; 43.7%), and Child–Pugh C (54 of 199; 27.1%). Ascites was the most frequent cause of decompensation, occurring in 135 patients (82.5%), whereas variceal bleeding occurred in 20 (14.0%) patients and hepatic encephalopathy in five (3.5%). Three out of 56 compensated cirrhotic patients (5.3%) developed decompensation during the follow-up, including ascites in two patients and variceal bleeding in one.

**Fig. 1:**
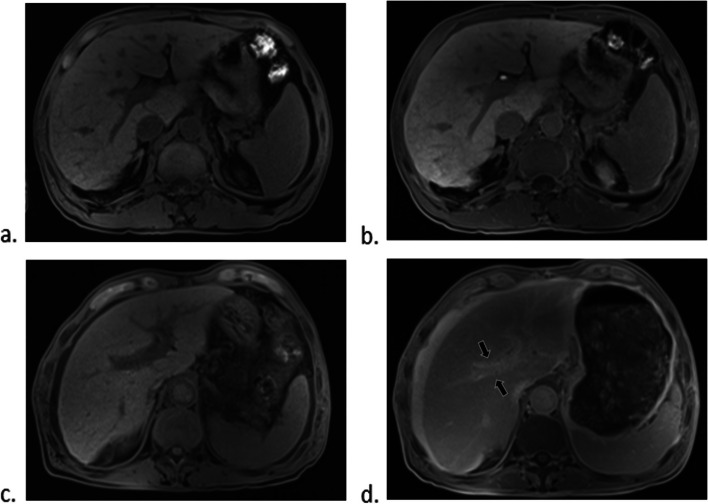
The pre-contrast T1-weighted image (**a**) and HBP image (**b**) were selected from a 57-year-old compensated cirrhotic patient. Periportal hyperintensity on HBP is not observed in this patient. The pre-contrast T1-weighted image (**c**) and HBP image (**d**) were selected from a 48-year-old decompensated cirrhotic patient. Periportal hyperintensity on HBP is observed (black arrow) in this patient. HBP, hepatobiliary phase

Periportal hyperintensity sign was observed in 21 patients (10.5%) on HBP. The interobserver agreement of the presence of periportal hyperintensity sign on HBP was excellent (*κ*  =  0.884 [95% CI  =  0.772, 0.995]). There was a significant difference in the frequency of periportal hyperintensity sign on HBP between compensated and decompensated cirrhotic patients (*p*  <  0.05) (Table 1, Fig. 1). The MELD score and serum liver function parameters, including AST, ALT, and total bilirubin, were significantly higher in patients with periportal hyperintensity sign on HBP than in those without the sign (*p*  <  0.05) (Table 2).

**Table 1:** Comparison of baseline characteristics in control and decompensated cirrhosis groups

	All (*n* = 199)	Control group (*n* = 56)	Decompensated cirrhosis group (*n* = 143)	*p* value
Sex				0.043
Male	135 (67.8%)	44 (78.6%)	91 (63.6%)	
Female	64 (32.2%)	12 (21.4%)	52 (36.4%)	
Age (years)	51 (45–60)	48 (44–57)	51 (45–61)	0.129
Cause of cirrhosis				0.089
Hepatitis B virus	137 (68.8%)	44 (78.6%)	93 (65.0%)	
Alcohol use	21 (10.6%)	2 (3.6%)	19 (13.3%)	
Cryptogenic disease	14 (7.0%)	4 (7.1%)	10 (7.0%)	
Autoimmune liver disease	11 (5.5%)	1 (1.8%)	10 (7.0%)	
Cholestasis	7 (3.5%)	1 (1.8%)	6 (4.2%)	
Hepatitis C virus	5 (2.5%)	1 (1.8%)	4 (2.8%)	
Drug use	4 (2.0%)	3 (5.4%)	1 (0.7%)	
Aspartate aminotransferase (U/L)	58.0 (35.0–89.0)	45.5 (33.5–81.5)	61.0 (36.0–91.0)	0.244
Alanine transaminase (U/L)	43.0 (26.0–83.0)	51.5 (29.0–131.0)	41.0 (25.0–76.0)	0.043
Total bilirubin (μmol/L)	33.1 (20.9–75.7)	25.8 (18.3–56.4)	38.4 (24.9–78.7)	0.011
Albumin (g/L)^a^	34.2 ± 5.8 (16.4–62.7)	37.1 ± 5.3 (26.6–50.3)	33.1 ± 5.7 (16.4–62.7)	< 0.0001
Creatinine (μmol/L)	62.5 (53.0–75.0)	63.5 (51.3–74.8)	62.0 (53.0–75.0)	0.948
PT (s)	14.6 (13.0–16.8)	13.6 (12.7–14.5)	15.5 (13.4–17.8)	< 0.0001
INR	1.3 (1.1–1.5)	1.1 (1.0–1.2)	1.3 (1.1–1.5)	< 0.0001
Child–Pugh class				< 0.0001
A	58 (29.1%)	35 (62.5%)	23 (16.1%)	
B	87 (43.7%)	20 (35.7%)	67 (46.9%)	
C	54 (27.1%)	1 (1.8%)	53 (37.1%)	
MELD score^a^	9 ± 5 (-3 to 34)	7 ± 5 (-1 to 18)	10 ± 5 (-3 to 34)	0.001
Periportal hyperintensity sign on HBP	21 (10.5%)	2 (3.5%)	19 (13.2%)	0.045

Data are presented as median (interquartile range) or data (percentage)

*PT* prothrombin time, *INR* international normalized ratio, *MELD* model for end-stage liver disease

^a^Data are means ± standard deviation with ranges in parentheses

**Table 2:** Serum parameters in patients with and without periportal hyperintensity sign on HBP MRI

	With periportal hyperintensity sign on HBP (*n* = 21)	Without periportal hyperintensity sign on HBP (*n* = 178)	*p* value
Aspartate aminotransferase (U/L)	83.0 (54.0–131.5)	54.0 (34.0–81.0)	0.002
Alanine transaminase (U/L)	77.0 (39.0–132.5)	40.0 (25.0–78.2)	0.006
Total bilirubin (μmol/L)	99.4 (44.4–163.1)	30.4 (20.5–69.6)	< 0.0001
Albumin (g/L)^a^	36.1 ± 7.6 (27.3–62.7)	34.0 ± 5.6 (16.4–52.7)	0.131
Creatinine (μmol/L)	63.8 (52.3–73.7)	62.4 (53.4–75.0)	0.966
PT (s)	15.5 (12.1–17.7)	14.5 (13.1–16.8)	0.930
INR	1.3 (1.1–1.5)	1.2 (1.1–1.4)	0.397
MELD score^a^	12.6 ± 6.3 (6–34)	9.0 ± 5.0 (-3 to 23)	0.002

Data are presented as median (interquartile range) or data (percentage)

*PT* prothrombin time, *INR* international normalized ratio, *MELD* model for end-stage liver disease

^a^Data are means ± standard deviation with ranges in parentheses

### The value of periportal hyperintensity sign on HBP MRI for predicting death in patients with decompensated cirrhosis

After a median follow-up of 29.0 months (range, 1.0–90.0 months), 9 out of 143 patients with decompensated cirrhosis (6.2%) died. In the univariate analysis, periportal hyperintensity sign on HBP MRI (HR  =  28.056; 95% CI  =  5.808–135.524; *p*  <  0.0001) and MELD score (HR  =  1.184; 95% CI  =  1.078–1.302; *p*  =  0.0004) were associated with death. In the multivariate analysis, periportal hyperintensity sign on HBP (HR  =  23.677; 95% CI  =  4.759–117.788; *p*  =  0.0001) and MELD score (HR  =  1.140; 95% CI  =  1.037–1.252; *p*  =  0.006) were significant risk factors for death (Table 3).

**Table 3:** Cox survival analysis for predicting death in patients with decompensated cirrhosis

Variables	Univariate			Multivariate		
	HR	95% CI	*p* value	HR	95% CI	*p* value
Age (years)	1.025	0.964–1.090	0.427			
Sex (male)	1.386	0.372–5.160	0.627			
Periportal hyperintensity sign on HBP	28.056	5.808–135.524	< 0.0001	23.677	4.759–117.788	0.0001
Periportal hyperintensity sign on T2WI	1.413	0.177–11.300	0.745			
Aspartate aminotransferase (U/L)	1.000	0.990–1.010	0.933			
Alanine transaminase (U/L)	0.999	0.992–1.007	0.868			
Albumin (g/L)	1.040	0.947–1.143	0.413			
PT	1.035	0.944–1.135	0.461			
MELD score	1.184	1.078–1.302	0.0004	1.140	1.037–1.252	0.006

*HR* hazard ratio, *CI* confidence interval, *PT* prothrombin time, *INR* international normalized ratio, *MELD* model for end-stage liver disease

**Fig. 2:**
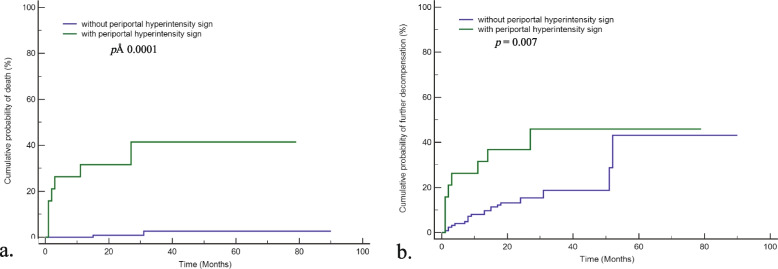
Kaplan–Meier curves for patients with decompensated cirrhosis. **a** Cumulative incidence of death in patients with periportal hyperintensity sign on HBP compared to those without the sign. **b** Cumulative incidence of further decompensation in patients with periportal hyperintensity sign on HBP compared to those without the sign. HBP, hepatobiliary phase

The AUC of periportal hyperintensity sign on HBP MRI was 0.844 (95% CI  =  0.774–0.899). Decompensated cirrhotic patients with periportal hyperintensity sign on HBP MRI had a significantly higher risk of death than those without the sign (*p*  <  0.0001) (Fig. 2a). The risk of death in patients with periportal hyperintensity sign on HBP MRI at 1, 3, and 5 years was 31.5%, 36.8%, and 36.8%, respectively. The risk of death in patients without periportal hyperintensity sign on HBP MRI at 1, 3, and 5 years was 0.8%, 1.6%, and 1.6%, respectively.

### The value of periportal hyperintensity sign on HBP for predicting further decompensation in patients with decompensated cirrhosis

After the follow-up, 30 out of 143 patients (20.9%) with decompensated cirrhosis progressed to further decompensation, with variceal bleeding being the most common presentation (*n*  =  12, 40.0%), followed by HE (*n*  =  11, 36.6%), liver-related death (*n*  =  9, 30.0%), and ascites (*n*  =  6, 20.0%). Multivariate analysis results showed that periportal hyperintensity sign on HBP (HR  =  2.594; 95% CI  =  1.140–5.903; *p*  =  0.023) and MELD score (HR  =  1.073; 95% CI  =  1.003–1.149; *p*  =  0.042) were significant risk factors of further decompensation (Table 4).

**Table 4:** Cox survival analysis for predicting further decompensation in patients with decompensated cirrhosis

Variables	Univariate			Multivariate		
	HR	95% CI	*p* value	HR	95% CI	*p* value
Age (years)	1.016	0.982–1.052	0.353			
Sex (male)	0.755	0.345–1.654	0.483			
Periportal hyperintensity sign on HBP	2.895	1.287–6.513	0.010	2.594	1.140–5.903	0.023
Periportal hyperintensity sign on T2WI	0.461	0.205–1.037	0.061			
Aspartate aminotransferase (U/L)	0.996	0.988–1.004	0.322			
Alanine transaminase (U/L)	0.997	0.998–1.005	0.410			
Albumin (g/L)	0.968	0.905–1.035	0.337			
PT	0.987	0.903–1.079	0.776			
MELD score	1.082	1.011–1.158	0.022	1.073	1.003–1.149	0.042

*HR* hazard ratio, *CI* confidence interval, *PT* prothrombin time, *INR* international normalized ratio, *MELD* model for end-stage liver disease

Decompensated cirrhotic patients with periportal hyperintensity sign on HBP had a significantly higher risk of further decompensation than those without the sign (*p*  =  0.007) (Fig. 2b). Additionally, the risk of further decompensation in patients with periportal hyperintensity sign on HBP at 1, 3, and 5 years was 31.6%, 36.8%, and 42.1%, respectively. The risk of further decompensation in patients without periportal hyperintensity sign on HBP at 1, 3, and 5 years was 8.1%, 16.1%, and 17.4%, respectively.

## Discussion

In the present study, there was a significant difference in the frequency of periportal hyperintensity sign on HBP between compensated and decompensated cirrhotic patients. Moreover, periportal hyperintensity sign from Gd-BOPTA-enhanced HBP MRI was a significant risk factor for death and further decompensation in patients with decompensated cirrhosis.

The timing of liver uptake and excretion of Gd-BOPTA begins at 40 min after injection and can last until 120 min at the hepatobiliary phase. In clinical practice, it is widely accepted that 90 min was a feasible liver uptake and excretion time point [13, 23]. Our study found that there was a significant difference in the frequency of periportal hyperintensity sign on HBP MRI between the control group and decompensated cirrhosis group, suggesting that periportal hyperintensity sign on HBP MRI may distinguish decompensated cirrhotic patients from compensated cirrhotic patients. The reason might be that patients with decompensated cirrhosis have poor liver function, resulting in a poor enhancement of liver parenchyma and a relative increase of signal intensity around the portal vein [17, 24]. Ascites is the most common complication in patients with decompensated cirrhosis [25]. As stated by Ciolina et al. [26], pleural and peritoneal fluids appear hyper/isointense on HBP MRI in most patients after Gd-BOPTA injection, while fluids remain hypointense on HBP MRI after gadoxetate disodium (Gd-EOB-DTPA) injection.

As the risk of death in decompensated cirrhotic patients is about four to five times higher than that in compensated cirrhotic patients [27], early identification and treatment are crucial for patients who are at higher risk of death. In the present study, periportal hyperintensity sign on HBP MRI was a significant risk factor for death in patients with decompensated cirrhosis. Several studies about periportal hyperintensity on HBP MRI have been done. Lampichler et al. [22] found that periportal hyperintensity sign on HBP MRI may correspond to active inflammation or periportal fibrosis. Zheng et al. [28] demonstrated that periportal hyperintensity sign on HBP MRI can be useful for predicting advanced liver fibrosis. As liver fibrosis accumulates and inflammation activity increases, liver function deteriorates rapidly [29]. The fact that liver function determines patient survival could have accounted for our finding [6, 30]. The identification of patients with periportal hyperintensity sign would be invaluable in deciding when and in which patients to intensify medical care. It could also aid in selecting candidates for liver transplantation, thereby facilitating focused resource allocation by identifying those at high risk of death [31]. In addition, the MELD score was also a significant risk factor for death in patients with decompensated cirrhosis in this study. The MELD score may play an important role in evaluating clinical outcomes of patients with decompensated cirrhosis, which is similar to previous studies [6, 25].

The present study also showed that periportal hyperintensity sign on HBP MRI was a significant risk factor for further decompensation in patients with decompensated cirrhosis. These results may be explained by the persistent inflammatory state in these patients, which may easily trigger further decompensation [25, 32]. Therefore, periportal hyperintensity sign on HBP MRI may be useful for clinicians to pursue relevant interventions in order to prevent further decompensation and to stabilize the disease progression. Additionally, the finding that the MELD score was associated with further decompensation is consistent with that described by a previous study by Zanetto et al. [21].

There were several limitations in the study. First, this was a retrospective, single-center study, which may introduce inherent selection bias. Second, clinical records were the principal source of information on cirrhosis diagnosis and not all cases were histopathologically confirmed [17]. Third, the sample size of patients with periportal hyperintensity sign was small [24]. Therefore, further clinical trials in larger populations are necessary in order to validate the present study findings.

In conclusion, periportal hyperintensity sign from Gd-BOPTA-enhanced HBP MRI is valuable for predicting clinical outcomes in patients with decompensated cirrhosis. This information might be helpful for clinicians to guide treatment and eventually improve clinical outcomes.

## Data Availability

The datasets used and/or analyzed during the current study are available from the corresponding author upon reasonable request.

## Abbreviations

ALT: Alanine aminotransferase
AST: Aspartate aminotransferase
AUC: Area under the curve
CI: Confidence interval
Gd-BOPTA: Gadobenate dimeglumine
Gd-EOB-DTPA: Gadoxetate disodium
HBP: Hepatobiliary phase
HE: Hepatic encephalopathy
HR: Hazard ratio
MELD: Model for end-stage liver disease
ROC: Receiver operating characteristic

## References

[1] Jang JW, Choi JY, Kim YS (2018). Effects of virologic response to treatment on short- and long-term outcomes of patients with chronic hepatitis B virus infection and decompensated cirrhosis. Clin Gastroenterol Hepatol.

[2] de Jongh FE, Janssen HL, de Man RA, Hop WC, Schalm SW, van Blankenstein M (1992). Survival and prognostic indicators in hepatitis B surface antigen-positive cirrhosis of the liver. Gastroenterology.

[3] European Association for the Study of the Liver (2018). Electronic address: easloffice@easloffice.eu; European Association for the Study of the Liver. EASL Clinical Practice Guidelines for the management of patients with decompensated cirrhosis. J Hepatol..

[4] Jang JW, Choi JY, Kim YS (2015). Long-term effect of antiviral therapy on disease course after decompensation in patients with hepatitis B virus-related cirrhosis. Hepatology.

[5] Langberg KM, Kapo JM, Taddei TH (2018). Palliative care in decompensated cirrhosis: a review. Liver Int.

[6] Zipprich A, Garcia-Tsao G, Rogowski S, Fleig WE, Seufferlein T, Dollinger MM (2012). Prognostic indicators of survival in patients with compensated and decompensated cirrhosis. Liver Int.

[7] Pugh RN, Murray-Lyon IM, Dawson JL, Pietroni MC, Williams R (1973). Transection of the oesophagus for bleeding oesophageal varices. Br J Surg.

[8] Kang SH, Jeong WK, Baik SK, Cha SH, Kim MY (2018). Impact of sarcopenia on prognostic value of cirrhosis: going beyond the hepatic venous pressure gradient and MELD score. J Cachexia Sarcopenia Muscle.

[9] Butt AA, Ren Y, Lo Re V 3rd, Taddei TH, Kaplan DE (2017) Comparing Child-Pugh, MELD, and FIB-4 to predict clinical outcomes in hepatitis C virusinfected persons: results from ERCHIVES. Clin Infect Dis 65(1):64–72 10.1093/cid/cix224 28369305

[10] Christensen E (2004). Prognostic models including the Child-Pugh, MELD and Mayo risk scores–where are we and where should we go?. J Hepatol.

[11] Durand F, Valla D (2005). Assessment of the prognosis of cirrhosis: Child-Pugh versus MELD. J Hepatol..

[12] Bonatti M, Valletta R, Avesani G (2021). Liver enhancement during hepatobiliary phase after Gd-BOPTA administration: correlation with liver and renal function. Eur Radiol.

[13] Liu C, Sun Y, Yang Y (2021). Gadobenate dimeglumine-enhanced biliary imaging from the hepatobiliary phase can predict progression in patients with liver cirrhosis. Eur Radiol.

[14] Zhao X, Huang M, Zhu Q, Wang T, Liu Q (2015). The relationship between liver function and liver parenchymal contrast enhancement on Gd-BOPTA-enhanced MR imaging in the hepatocyte phase. Magn Reson Imaging.

[15] Cai S, Lin N, Yang Y (2023). The value of contrast-enhanced portal vein imaging at the hepatobiliary phase obtained with gadobenate dimeglumine for predicting decompensation and transplant-free survival in chronic liver disease. Eur Radiol.

[16] Ly JN, Miller FH (2001). Periportal contrast enhancement and abnormal signal intensity on state-of-the-art MR images. AJR Am J Roentgenol.

[17] Kobayashi S, Matsui O, Gabata T (2013). Intrahepatic periportal high intensity on hepatobiliary phase images of Gd-EOB-DTPA-enhanced MRI: imaging findings and prevalence in various hepatobiliary diseases. Jpn J Radiol.

[18] Ripoll C, Groszmann R, Garcia-Tsao G (2007). Hepatic venous pressure gradient predicts clinical decompensation in patients with compensated cirrhosis. Gastroenterology.

[19] Scheiner B, Steininger L, Semmler G (2019). Controlled attenuation parameter does not predict hepatic decompensation in patients with advanced chronic liver disease. Liver Int.

[20] Jachs M, Hartl L, Simbrunner B (2022). Decreasing von Willebrand factor levels upon nonselective beta blocker therapy indicate a decreased risk of further decompensation, acute-on-chronic liver failure, and death. Clin Gastroenterol Hepatol.

[21] Zanetto A, Campello E, Bulato C (2022). Increased platelet aggregation in patients with decompensated cirrhosis indicates higher risk of further decompensation and death. J Hepatol.

[22] Lampichler K, Semmler G, Wöran K (2022). Imaging features facilitate diagnosis of porto-sinusoidal vascular disorder. Eur Radiol.

[23] Lebert P, Adens-Fauquembergue M, Azahaf M (2019). MRI for characterization of benign hepatocellular tumors on hepatobiliary phase: the added value of in-phase imaging and lesion-to-liver visual signal intensity ratio. Eur Radiol.

[24] Onishi H, Theisen D, Zachoval R, Reiser MF, Zech CJ (2019). Intrahepatic diffuse periportal enhancement patterns on hepatobiliary phase gadoxetate disodium-enhanced liver MR images: Do they correspond to periportal hyperintense patterns on T2-weighted images?. Medicine (Baltimore).

[25] Balcar L, Tonon M, Semmler G (2022). Risk of further decompensation/mortality in patients with cirrhosis and ascites as the first single decompensation event. JHEP Rep.

[26] Ciolina M, Di Martino M, Bruno O, Pommier R, Vilgrain V, Ronot M (2018). Peritoneal and pleural fluids may appear hyperintense on hepatobiliary phase using hepatobiliary MR contrast agents. Eur Radiol.

[27] Malik A, Kumar D, Khan AA (2018). Hepatitis B virus precore G1896A mutation in chronic liver disease patients with HBeAg negative serology from North India. Saudi J Biol Sci.

[28] Zheng W, Guo W, Xiong M (2023). Clinic-radiological features and radiomics signatures based on Gd-BOPTA-enhanced MRI for predicting advanced liver fibrosis. Eur Radiol.

[29] Feier D, Balassy C, Bastati N, Stift J, Badea R, Ba-Ssalamah A (2013). Liver fibrosis: histopathologic and biochemical influences on diagnostic efficacy of hepatobiliary contrast-enhanced MR imaging in staging. Radiology.

[30] Tian M, Liu W, Tao C (2020). Prediction of overall survival in resectable intrahepatic cholangiocarcinoma: ISICC-applied prediction model. Cancer Sci.

[31] Angeli P, Rodríguez E, Piano S (2015). Acute kidney injury and acute-on-chronic liver failure classifications in prognosis assessment of patients with acute decompensation of cirrhosis. Gut.

[32] Gupta A, Rana R, Agarwal S (2023). Assessing the risk of further decompensation and survival in patients with cirrhosis with variceal bleeding as their first decompensation event. Am J Gastroenterol.

