# Correction: The value of periportal hyperintensity sign from gadobenate dimeglumine-enhanced hepatobiliary phase MRI for predicting clinical outcomes in patients with decompensated cirrhosis

**DOI:** 10.1186/s13244-024-01706-8

**Published:** 2024-05-12

**Authors:** Lanqing Cong, Yan Deng, Shuo Cai, Gongzheng Wang, Xinya Zhao, Jingzhen He, Songbo Zhao, Li Wang

**Affiliations:** 1 Department of Radiology, Shandong Provincial Hospital Affiliated to Shandong First Medical University, Jinan, Shandong Province China; 2 Department of Radiology, Qilu Hospital of Shandong University, Jinan, Shandong China; 3 MRI Department, Shandong Provincial Hospital Heze Hospital, Heze, Shandong China; 4 Department of Radiology, Shandong Provincial Hospital, Shandong University, Jinan, Shandong China; 5 Department of Central Laboratory, Shandong Provincial Hospital Affiliated to Shandong University, Jinan, Shandong China; 6 Shandong Provincial Engineering and Technological Research Center for Liver Diseases Prevention and Control, Jinan, Shandong China; 7 Department of Health Management Center, Shandong Provincial Hospital Affiliated to Shandong First Medical University, Jinan, Shandong China

**Correction to:** **Insights into Imaging (2024)** **15:64** 10.1186/s13244-024-01629-4

The authors wish to note that in Figure 2a of the original article, the special symbol should instead be “<”.

